# Health and social care staff’s recognition of elder abuse perpetrated by family members of persons with dementia: a mixed-method study

**DOI:** 10.1177/14034948241261724

**Published:** 2024-08-09

**Authors:** Linda Johansson, Jonas Sandberg, Marie Ernsth Bravell, Lena Östlund

**Affiliations:** 1 Institute of Gerontology, School of Health and Welfare, Jönköping University, Jönköping, Sweden; 2 Department of Nursing Science, Sophiahemmet University, Stockholm, Sweden; 3 Dean’s Office, School of Health and Welfare, Jönköping University, Jönköping, Sweden

**Keywords:** Dementia, dental care, domestic violence, elder abuse, health personnel, intimate partner violence, prevention, Sweden

## Abstract

**Background:**

Health and social care staff play a significant role in detecting and reporting abuse among persons with dementia. However, they are often left to their own judgements which can lead to elder abuse not being detected or acted on. The aim was to explore what healthcare and social care staff consider elder abuse, and their experience of elder abuse perpetrated by family members of persons with dementia.

**Methods:**

This mixed-method vignette study was conducted in Sweden during the year 2021. In total 39 staff working in dementia care were included. They first answered the Caregiver Scenario Questionnaire and then participated in a group interview.

**Results:**

An inconsistency was revealed regarding whether a management strategy for behavioural difficulties included in the Caregiver Scenario Questionnaire should be considered an abusive act or not. No participants were able to identify all five abusive behaviour management strategies. Participants described witnessing 101 abusive acts including different types of abuse of a person with dementia, with emotional/psychological abuse and neglect being most common.

**Conclusions:**

**Health and social care staff who work close to older persons are able to detect abuse perpetrated by family members. However, inconsistency in defining abusive acts demonstrates the uncertainty in identifying abuse. This may lead to abuse not being identified, but it also creates feelings of inadequacy among staff.**

## Introduction

Elder abuse is recognised as a global problem and according to a meta-analytic review paper, the pooled prevalence rate for overall elder abuse is 15.7% [1]. Being a victim of abuse is negatively associated with health and wellbeing, including premature mortality, increased utilisation of healthcare, and various forms of physical and mental symptoms such as depression, pain and sleeping problems [2]. In Sweden, there are national regulations [3] stating that especially social care staff, but also healthcare and dental care staff, should have knowledge to detect abuse and offer support when needed. Yet, a recent study showed that approximately 25% of older persons have experienced some form of abuse at any time during their life, but only 2% had ever been asked about violence victimisation in healthcare [4].

Elder abuse is commonly defined as a single or repeated act or lack of appropriate action, occurring within any relationship where there is an expectation of trust, which causes harm or distress to an older person [5, p. 2]. There are different types of elder abuse with physical, emotional/psychological, sexual, and financial abuse/exploitation as well as neglect being most common [5, 6] and these types are included in the current study. In the past decade, the concept of polyvictimisation has received more focus which can exacerbate the harm to the older person compared with being subjected to only one type of abuse [7]. Polyvictimisation occurs when there is harm through multiple co-occurring or sequential types of elder abuse by one or more perpetrators, or when an older adult experiences one type of abuse perpetrated by multiple others [8, p. 4].

One risk factor for being exposed to abuse among older persons is cognitive impairment. According to a recent literature review 0.3–78.4% of persons with dementia have experienced abuse of some kind [9]. The enormous difference in prevalence seems to be related to methodological issues such as the definition of abuse, the study context, and sampling techniques, as well as how abuse is measured, which affects the reporting of abuse [9, 10].

For victims with dementia disease, the abuser is often a family member [11]. One reason is that family members can become caregivers when a person with dementia becomes dependent on help from others to manage daily life, but may lack knowledge about how to care for the person with dementia. In addition, caregiver burden and stress are considered by some commentators as one of the most common risk factors for elder abuse [10]. However, stress and burden are not the only reasons for abuse. There are a number of other risk factors for becoming an abuser such as substance use, physical and mental health problems, and relationship problems [12]. Also, abuse could have existed before the cognitive impairment [13].

When being abused by a family member, it is not uncommon that the abused older person avoids reporting this due to fear of the consequences both for the abuser and themselves [14]. Also, persons with dementia are less likely to be considered credible when disclosing abuse [7], which might prevent them from reporting.

A shrinking social network is not uncommon when it comes to persons with dementia, and for some persons one of the closest and most trusting relationships they have is with health and/or social care staff. Consequently, staff in health and social care services play significant roles in detecting and reporting elder abuse. However, it can be difficult for health and social care staff to separate symptoms of elder abuse from normal aging, and several signs of abuse are similar to those of cognitive impairment [10]. This can result in staff having trouble recognising expressions of fear and anxiety, demonstrations of poor hygiene and malnutrition, or physical injuries such as bruises as signs of elder abuse [15]. All these challenges can make it difficult to detect abuse and give support to the victims. Using screening instruments can assist health and social care staff to identify risks of elder abuse in a structured way [16]. However, there is a lack of existing screening tools to detect abuse in persons with dementia [17].

In addition, lacking resources and guidelines leaves health and social care staff acting on their own judgement and experience [18]. Having poor awareness and knowledge regarding elder abuse negatively affects their likelihood to detect and report abuse [19]. Consequently, studying the nature of elder abuse and getting a more comprehensive picture of the phenomenon and its meaning for staff is a first step towards being able to develop suitable interventions in health and social care. Therefore, the aim of this study was to explore: (a) what health and social care staff consider abusive acts; and (b) notions of elder abuse perpetrated by family members of persons with dementia witnessed in their daily work.

## Methods

### Design

**Figure 1.**
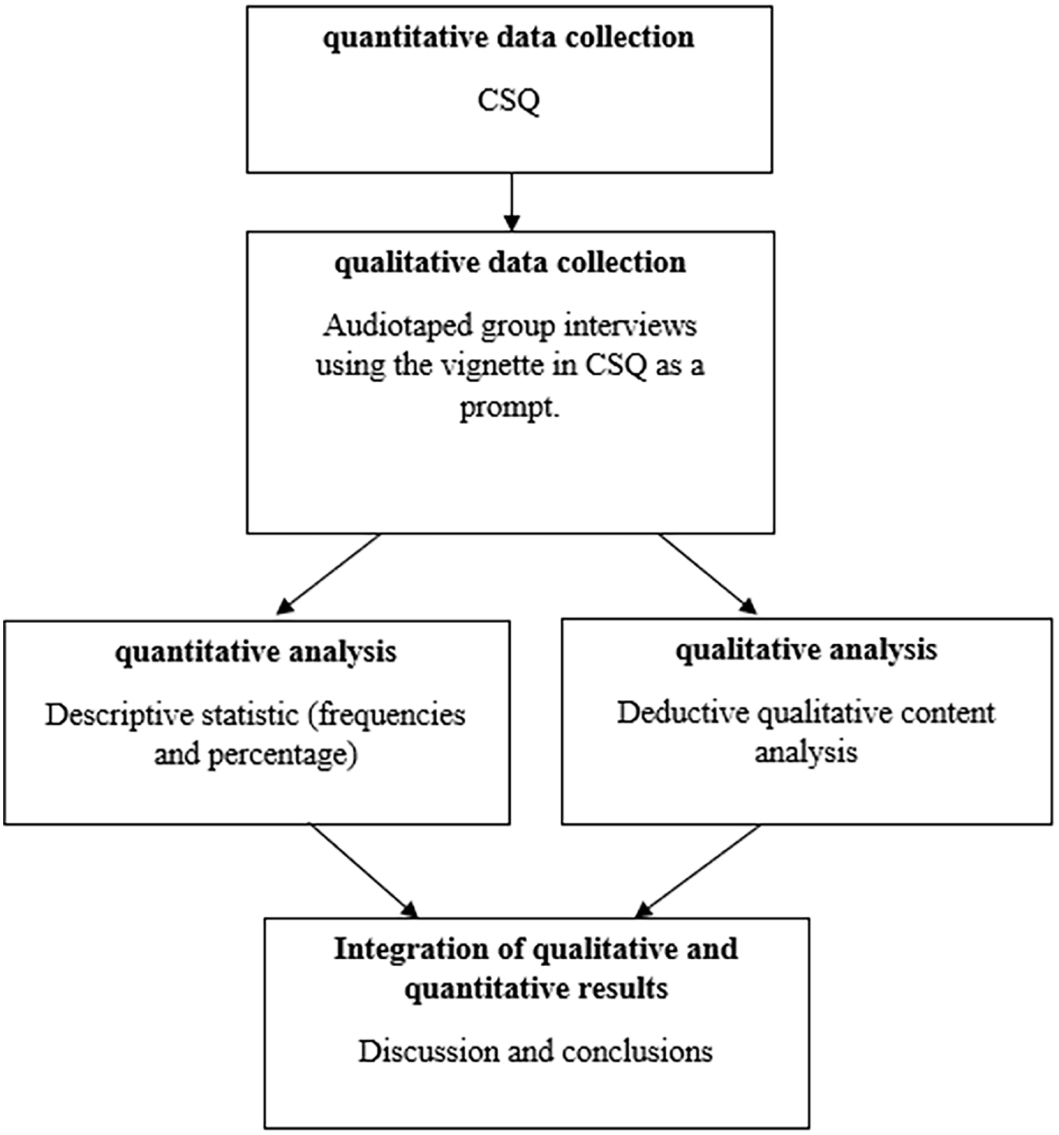
A visual description of the mixed-method design used in the study.

To provide a fuller picture and a comprehensive understanding of the phenomenon, a mixed-method sequential explanatory design was used in which the qualitative data were collected after the quantitative data [20]. A vignette technique was used when collecting data. Vignettes have earlier been used in both quantitative and qualitative studies. Vignettes are commonly used in social and nursing research, not least in studies focusing on potentially sensitive topics [21]. In the current study a fictional vignette is part of the Caregiver Scenario Questionnaire (CSQ) [22]. The quantitative part of the study is a descriptive analysis of the participants’ answers on the CSQ. Qualitative data were collected using group interviews in which the vignette in the CSQ was used as a prompt to discuss elder abuse in general and elder abuse that the participants had encountered in their professional role. Group interviews were chosen as they encourage participants to discuss and reflect on their thoughts and experiences with each other [23]. Quantitative and qualitative data were analysed and presented separately and then, as described by Ivankova et al. [20], results were integrated in the discussion and conclusion, as shown in Figure 1. The study was conducted during 2021 and data from the qualitative interviews have been used in another study aiming to explore how staff reason and act when suspecting domestic abuse in persons with dementia [18].

### Setting and participants

In Sweden, dementia care is a shared responsibility between the regions (*n*=20) and the municipalities (*n*=290). The regions are responsible for healthcare and dental care while the municipalities provide social care and healthcare (up to the level of nurses). Physicians are not employed by the municipalities. Participants were recruited from both regions and municipalities. A purposeful sampling approach was used, meaning that for inclusion staff should work close to older persons with dementia through examinations, treatment, care or assistance.

Managers in healthcare, dental care and social care helped identify and inform potential participants about the study. Physicians were informed of the study and invited to participate by the researchers when attending a specialist training course. In total, 39 staff working in healthcare (*n*=13), social care (*n*=21), and dental care (*n*=5) were included, with three to six staff participating in each group interview. In total, three municipalities and two regions were represented. The median age of the participants was 45.5 years (q1 34.0; q3 54.75), 32 persons (82.1%) were women and seven (17.9%) were men. Most participants (*n*=26, 66.7%) had a university degree. Further descriptions of the participants in the different groups are found in Table I.

**Table I.** Description of participants in the different groups (*n*=39).

Number of participants in each group	Types of care (employer)	Occupation (*n*)
5	Home healthcare (municipality)	Registered nurse (*n*= 4)
		Occupational therapist (*n*=1)
6	Home healthcare (municipality)	Registered nurse (*n*=2)
		Occupational therapist (*n*=2)
		Certified nursing assistant (*n*=1)
		Senior educator (*n*=1)
4	Home help service (municipality)	Certified nursing assistant (*n*=4)
6	Home help service (municipality)	Certified nursing assistant (*n*=1)
		First-line manager (*n*=1)
		Care assistant (*n*=4)
4	Hospital (region)	Physician (n=4)
5	Oral care (region)	Dentist (*n*=2)
		Dental hygienist (*n*=2)
		Dental nurse (*n*=1)
6	Memory clinic (region)	Registered nurse (*n*=2)
		Geriatrician (*n*=2)
		Occupational therapist (*n*=1)
		Social worker (*n*=1)
3	Primary care (region)	Registered nurse (*n*=2)
		General practitioner (*n*=1)

### Data collection

One week before each interview an email was sent to the participants including an information letter and the CSQ to be printed, answered and returned at the time of the interview. The CSQ was developed in the UK [22] and is a widely adopted [24–26] instrument for investigating the recognition of abusive acts among different professionals. For the current study we used version B of the CSQ [27], which includes a fictional vignette describing a potentially difficult situation for a couple in which the husband John has a dementia disease. The wife, Ulla, had earlier refused formal support and she wants John to continue living with her. However, she is now asking for advice as John’s cognition has deteriorated, which creates a stressful situation for the wife. Users of the CSQ are asked to evaluate whether different behaviour management strategies are abusive acts on a 6-point Likert scale including the following options: Good idea and helpful, Possibly useful, Not sure, Unlikely to help, Bad idea but not abusive (all these options are considered non-abusive in the dichotomised version) and Abusive [22, 27]. As there is no existing Swedish version of the questionnaire, a translation was provided by the researchers after permission from the developer of the CSQ. Three staff working in dementia care were asked for their opinions of the translated version of the CSQ before it was used in the study, which resulted in some minor revisions of the questionnaire. Later it was revealed that one of the strategies was incorrect as it was wrongly phrased in the original CSQ. Consequently, this item was excluded in this study so that the CSQ included 13 behaviour management strategies. In line with other studies using the CSQ [see for instance 22, 24–26], each strategy was categorised by the authors as ‘abusive’, ‘potentially abusive’ or ‘not abusive’. The categorisation was based on Swedish regulations and a judgement conducted by an expert panel.

In each of the eight group interviews (six face to face; two digitally performed using Zoom) a moderator and an assistant were present in accordance with the group interview technique. The moderator led the discussion using an interview guide including questions about perceptions of the presented vignette and management strategies in the CSQ, witnessing situations in daily work that were considered as abuse, and interventions when a person with dementia was exposed to abuse (Table II). The assistant was responsible for the technical equipment as well as summarising what had been said at the end of each interview. Each interview lasted approximately one hour, was audio-recorded and transcribed verbatim. The study received ethical approval from the Swedish Ethical Review Agency (reg. no. 2019-03031).

**Table II.** Interview guide.

1. What do you think about the vignette in the questionnaire? Is there any action you pondered on more? Is there any strategy you consider to be abuse or violence? 2. Was there anything in the vignette description that you recognise from your daily work? 3. Which experiences do you have of encountering older people with dementia who have been subjected to domestic abuse? 4. What leads you to suspect domestic abuse among persons with dementia? 5. How do you act in the encounter with abused persons with dementia and their relatives? 6. What tools do you have for detecting and taking action when domestic abuse in persons with dementia occurs?

### Data analysis

Descriptive statistics (frequencies and percentages) were used to illustrate participants’ perceptions of the behaviour management strategies in the CSQ. Chi-square analyses were performed for each item to assess consensus in the perceptions. For interview data, a deductive qualitative content analysis in line with the description of Elo and Kyngäs [28] was performed using a categorisation matrix that included different types of abuse, as defined by Hall et al. [6], see Table III. Only examples of witnessed elder abuse in which the victim was a person with dementia and the abuser was a close relative were included, and the number of identified examples were counted. A described situation could include several types of abuse, and was then included in all types identified, that is, one witnessed situation could be analysed as consisting of more than one type of abuse.

**Table III.** Matrix of types of elder abuse used in the deductive qualitative content analysis.

Types of abuse	Description of abuse^a^	Examples of witnessed acts	Total number of acts
Physical abuse	The intentional use of physical force that results in acute or chronic illness, bodily injury, physical pain, functional impairment, distress, or death. Physical abuse may include but is not limited to such acts of violence as striking (with or without an object or weapon), hitting, beating, scratching, biting, choking, suffocation, pushing, shoving, shaking, slapping, kicking, stomping, pinching, and burning. In addition, inappropriate use of medications and physical restraints, pinning in place, arm twisting, hair pulling, force-feeding, and physical punishment of any kind also are examples of physical abuse (page 31).	I have seen relatives get angry because we have not given the patient medication by force. They [person with dementia] don’t want to take the medication. And then the relatives come over, take the medication, and force it into the mouth of the mother, who tries to spit it out and says she doesn’t want it. And then she [the relative] says, ‘Look how easy it is, now she has taken her medicine. Why do I have to come here? Why don’t you make sure she gets her medicine?’ (i1) I thought I saw. . . when I checked the blood pressure and such, that he had bruises. (i8) The husband tells me how he sometimes gets so tired when she talks about having her keys with her, for example, when they are going somewhere ‘that I can throw the keys at her or after her’. And more and more [incidents] like this came up. (i6)	16
Emotional/ psychological abuse	Verbal or non-verbal behaviour that results in the infliction of anguish, mental pain, fear, or distress, that is perpetrated by a caregiver or other person who stands in a trust relationship to the elder. Such behaviours may have immediate effects or delayed effects that are short or long-term in nature that may or may not be readily apparent to or acknowledged by the victim. May include any of the following and vary according to cultural norms: Humiliation/disrespect Threats Harassment Isolation/coersive control (pages 33–34)	One time, I found it very sad, the grandson was visiting, and they were going outside to get pizza. The grandma and grandkid. But grandpa isn’t going to come along. I said, ‘but have you asked him if he wants to go out?’ ‘No, he doesn’t want to.’ But have you asked him? ‘No, he’s in bed.’ And they went out and sat on the lawn with a picnic rug and everything, while he sat inside by himself, and they’ve left him a little piece of pizza. (i4) And you walk through the house together and the person with dementia shuffles along, and how does the wife react then? [she might say] ‘But I told you, sit over there. . .’ That’s what can happen and what you hear in some way is, ‘don’t do that, do this.’ (i2) He wanted nothing to do with her, and she refused to leave. And then there was a restraining order. So yes, there were many disputes where she often called me and wanted to get to him. So, it was very intense. (i3) So, you’re sitting here [with the person and relatives], trying to make a plan. ‘But she’s going home!’ ‘Yes, but what do you want?’ we asked this individual. ‘But mother, now say what you said before, that you want to go home.’ ‘Yes, but hey, now I’m actually asking your mother.’ ‘Yes, but she only talks to me.’ ‘No, she doesn’t.’ And then [asking the individual again]; ‘what do you want?’ And then that look, you know, when they look at the daughter, and don’t know what to answer. (i3)	48
Sexual abuse	Forced and/or unwanted sexual interaction (touching and non-touching acts) of any kind with an older adult. This may include but is not limited to forced and/or unwanted completed or attempted contact between the penis and the vulva or the penis and the anus involving penetration, however slight; forced and/or unwanted contact between the mouth and the penis, vulva, or anus; forced and/or unwanted penetration of the anal or genital opening of another person by a hand, finger, or other object; forced and/or unwanted intentional touching, either directly or through the clothing, of the genitalia, anus, groin, breast, inner thigh, or buttocks; unwarranted, intrusive, and/or painful procedures in caring for genitals or rectal area; or forced and/or unwanted non-contact acts of a sexual nature such as forcing a victim to view pornographic materials, photographing an elder for sexual gratification, voyeurism and verbal or behavioural sexual harassment (page 32).		0
Financial abuse/exploitation	The illegal, unauthorised, or improper use of an older individual’s resources by a caregiver or other person in a trusting relationship, for the benefit of someone other than the older individual. This includes, but is not limited to, depriving an older individual of rightful access to, information about, or use of personal benefits, resources, belongings, or assets (page 35).	Yes, it has happened that they [the relatives] have taken pain medication from their relatives [the person with dementia] so that there has been no medication. (i1) No, this was a specific case where it was obvious. Another relative of the patient said that the son had taken his [the person with dementia’s] credit card and lives in [another city] and uses it there. So, it was pretty clear. (i6) Because when we write a shopping list, initially, we bought a lot of yoghurt, cookies, buns, a lot of. . . Now it is him [the relative] that writes [the list] and it’s just two clementines, one apple, one loaf of bread. Nothing more. Interviewer: You mean it’s that precise? Yes, I think there’s a limit that should not be exceeded. Unfortunately, many only think about money. Interviewer: Money, yes. Well, you have to consider. . . Just so that they will receive a larger inheritance when the parents pass away. Yes, that’s what I was just going to say, the less you. . . we waste, the more I [the relative] receive in the end. Money, it’s okay, but I feel their pension is actually enough for her to eat reasonably. So, relatives don’t need to decide on it. (i4)	14
Neglect	Failure by a caregiver or other person in a trust relationship to protect an elder from harm or the failure to meet needs for essential medical care, nutrition, hydration, hygiene, clothing, basic activities of daily living or shelter, which results in a serious risk of compromised health and/or safety, relative to age, health status, and cultural norms (page 34).	You were at someone’s home where the husband either didn’t understand or didn’t accept that the patient in question was almost too poorly or had become so demented that she didn’t know how to eat a meal. And then he just accepted it when she said no, that she didn’t want to eat. (i8) She [the wife] didn’t want to brush [his teeth], but at the same time, she didn’t want him to move to a nursing home. No. So, they were both in the room, and I got to talk to her because he really needed help, but she didn’t want the home help service (i7) So, it’s usually been the case that you receive strong signals from the nurses, nursing assistants who help with personal care. That they have reactions like ‘here’s something that doesn’t make sense at all //’ For example, a very easy-to-manage patient who cooperates very well, and on the ward, the condition is improving very well. [A patient], who was previously developing pressure ulcers has now been able to get rid of them, or they have been able to be looked after very well. (i5)	23

a As defined by Hall et al. [6].

## Results

In total, 38 of the 39 participants answered the CSQ. The responses to the CSQ are found in Table IV. One participant (2.6%) did not identify any abusive behaviour management strategy correctly, six (15.8%) identified one strategy, 11 (28.9%) identified two strategies, eight (21.1%) identified three strategies, 12 (31.6%) identified four strategies, and none identified all five strategies correctly. ‘Ask their son to hold him down while she showered him’ was the strategy most often referred to as an abusive act (78.4%). All participants identified the non-abusive behaviour management strategies correctly according to the described types of abuse by World Health Organization [5] and Hall et al. [6]. Most participants (ranging between 94.1% and 100.0%) considered the possibly abusive behaviour management strategies as non-abusive, but there was lack of consistency regarding whether these strategies were useful or not. There were significant differences in the ratings in all but one strategy (Table IV).

**Table IV.** Staff’s assessments of suggested strategies for managing behaviours, *n* (%).

Strategy	Good idea and helpful	Possibly useful	Not sure	Unlikely to help	Bad idea but not abusive	Chi-square (*P*)	Summed: not abusive	Abusive
Abusive								
Ask their son to hold him down while she showers him (*n*=37)	0 (0.0)	2 (5.4)	2 (5.4)	2 (5.4)	2 (5.4)	78.8 (<0.001)	8 (21.6)	29 (78.4)
Lock him in the house while she goes shopping (*n*=37)	0 (0.0)	1 (2.7)	3 (8.1)	1 (2.7)	10 (27.0)	43.4 (<0.001)	15 (40.5)	22 (59.5)
Tell him that he cannot watch any television until he has had a wash (*n*=38)	0 (0.0)	5 (13.2)	4 (10.5)	15 (39.5)	11 (28.9)	14.1 (0.007)	35 (92.1)	3 (7.9)
Hide the sedative tablets in his morning cereal or tea (*n*=38)	2 (5.3)	7 (18.4)	2 (5.3)	1 (2.6)	4 (10.5)	50.1 (<0.001)	16 (42.1)	22 (57.9)
Push him back into his chair when he is hitting her so he cannot continue doing so (*n*=38)		2 (5.3)	4 (10.5)	2 (5.3)	6 (15.8)	45.7 (<0.001)	14 (36.8)	24 (63.2)
Possibly abusive								
Arrange for John to carry a mobile phone with GPS tracker so she can find him if he gets lost (*n*=38)	18 (47.4)	13 (34.2)	2 (5.3)	4 (10.5)	0 (0.0)	29.6 (<0.001)	37 (97.4)	1 (2.6)
Accept he refuses help attending to his personal hygiene needs even if this means he is unclean (*n*=34)	0 (0.0)	10 (29.4)	4 (11.8)	3 (8.8)	15 (44.1)	18.1 (0.001)	32 (94.1)	2 (5.9)
Tell him that if things continue the way they are going then he will have to live elsewhere (*n*=38)	0 (0.0)	5 (13.2)	2 (5.3)	21 (55.3)	10 (26.3)	22.0 (<0.001)	38 (100.0)	0 (0.0)
Lock herself in bedroom for periods when he is asking repeatedly to go out, so she does not have to keep answering (*n*=37)	0 (0.0)	3 (8.1)	2 (5.4)	12 (32.4)	18 (48.6)	28.5 (<0.001)	35 (94.6)	2 (5.4)
Not abusive								
Arrange for telecare so that he hears a recorded message of her voice telling him to go back to bed if he opens the front door at night (*n*=37)	4 (10.8)	9 (24.3)	9 (24.3)	11 (29.7)	4 (10.8)	5.57 (0.234)	37 (100)	0 (0.0)
Contact local services to request day care because he is not safe to be left alone (*n*=37)	35 (94.6)	2 (5.4)	0 (0.0)	0 (0.0)	0 (0.0)	29.4 (<0.001)	37 (100.0)	0 (0.0)
Request advice from nurses specialised in psychiatry or dementia on how to manage his aggression (*n*=38)	34 (89.5)	4 (10.5)	0 (0.0)	0 (0.0)	0 (0.0)	23.7 (<0.001)	38 (100.0)	0 (0.0)
Keep a diary of his behaviour for a few days to see whether there are certain times of day that are better for him to be washed (*n*=38)	28 (73.7)	9 (23.7)	0 (0.0)	1 (2.6)	0 (0.0)	30.4 (<0.001)	38 (100.0)	0 (0.0)

The deductive analysis focused on abuse against persons with dementia and revealed that participants had witnessed all forms of abuse committed by family members except for sexual abuse (*n*=0). The type of abuse, examples of witnessed abusive acts, and the number of acts are presented in Table III and below.

The most frequently observed acts concerned emotional/psychological abuse (*n*=48). These kinds of abusive acts were often verbal, meaning that they consisted of verbally attacking, insulting, humiliating, yelling at and/or threatening the person with dementia. Emotional/psychological abuse also included social isolation, controlling the person and refusing to let them leave. Family members sometimes also overstimulated the person with dementia and thought the person with dementia could manage more than they really could, which led to stress and indignity in the persons with dementia. Another part of the emotional/psychological abuse focused on violation of the person’s right to self-determination. Participants described witnessing how family members talked over the person’s head and made decisions against the person’s will. Observed physical abuse (*n*=16) included forcing the older person to take medicine or hiding medication in food and beverages, or that the person with dementia had been hit. Restraints were also described such as using belts or physically restraining the person. Sometimes the physical abuse itself was not directly witnessed, but participants described situations when they had observed symptoms of possible physical abuse such as bruises and bone fractures. When describing observed financial abuse/exploitation (*n*=14), participants mentioned abusive acts in which the person with dementia had been stolen from, including medications and money. Also, family members had taken over property from the person with dementia or had forced them to sign legal documents. Participants stated that they had witnessed persons with dementia not receiving the care they needed, for example meal support, because family members refused to pay for it. Sometimes it was difficult to know if this was a deliberate act or based on a lack of adequate financial resources. Neglect was one of the most witnessed types of abusive acts (*n*=23) meaning that family members sometimes failed to provide proper care for the person with dementia. Sometimes this was because the family members did not accept help from others, but were unable to manage the situation themselves. In other cases, participants explained that family members sometimes simply accepted the wishes of the person with dementia even though this led to inadequate care. Examples of witnessed neglect also included not taking care of the person’s medication treatment, hygiene, or nutritional needs.

## Discussion

No consensus was found in the participants’ assessments of the behaviour management strategies suggested in the CSQ regarding whether they were abusive acts or not. In relation to the categorisation done for this study, no participants were able to identify all five abusive strategies, with ‘Tell him that he cannot watch any television until he has had a wash’ being least commonly identified (7.9%). The findings from the CSQ highlight the complexity involved in identifying abusive acts, which is in line with studies from other countries using the CSQ [24, 25], as well as the results from the group interviews. Also, the expert panel highlighted that the strategy of not allowing John to watch television before washing was tricky to classify. They concluded that it could be considered as a threat and thereby should be assessed as emotional/psychological abuse. Whether or not a behaviour management strategy was considered abusive or not also seems to be related to the interpretation of the current context and situation, which not least was highlighted in the possibly abusive acts. In Sweden measures or strategies used without consent from the individual are considered coercion, including restraints even if the intention is to protect the person. This is also true for persons with dementia, even though they might lack the capacity to express and give informed consent [29]. Not defining and assessing abusive acts in a consistent way can lead to problems in detecting and thereby also responding to elder abuse in health and social care. However, studies have shown that training activities about elder abuse seem to be effective for helping staff to identify abusive acts better [27]. The present study also highlights the need for using screening tools as these can be helpful to identify and detect elder abuse in a more standardised and less subjective way. However, further research in this area is needed, and as elder abuse is complex and dependent on multiple factors it seems difficult to develop a universal instrument to assess all the aspects of abuse. Also, existing instruments should only be understood as screening instruments, meaning that results indicating elder abuse require more thorough assessments [30].

Even though participants had difficulties in identifying abusive behaviour management strategies in the CSQ, the interviews revealed testamonies of witnessing family members’ abusive acts towards persons with dementia. However, the uncertainty of what constitutes an abusive act means that participants may have missed recognising abusive situations. The numbers might therefore be even higher than the 101 identified acts in the current study. Health and social care staff’s own and earlier experiences might affect what they perceive as abuse [18] and thereby what they identify as abuse.

Emotional/psychological abuse as well as neglect were the most frequently described forms of abuse. In a Swedish interview study in which persons with cognitive impairment were excluded, older persons themselves also revealed that patterns of neglect and psychological abuse were the most prominent [31]. These types of abuse also seem to be among those most commonly identified in prevalence studies, together with physical abuse, even though there are huge variations in reported rates [9, 10]. However, measuring financial abuse and sexual abuse in research studies is less common, which might affect the prevalence rates [9]. No sexual abuse was described in the current study. However, it is not possible to know if this is a result of sexual abuse only occurring rarely, or if this is a result of staff having trouble identifying and recognising sexual abuse. A reason for the latter can be related to stereotypes around older persons and sexuality. This assumption of asexuality risks hiding the occurrence of sexual abuse and needs to be addressed further in the care of older persons.

Neglect included acts in which family members had not provided proper care and support regarding for instance nutrition, hygiene, and medication treatment. An interesting finding was that participants reported that family caregivers sometimes refused offered support despite finding it difficult to care for the person with dementia themselves. Participants reasoned that this was related to the family members not wanting to pay for support, but it was difficult to know if this was an intentional or unintentional act. This raises questions about the difficulties of distinguishing between intentional and unintentional abuse. However, being able to recognise if an act is intentional and unintentional can be important as it might affect what kind of support should be offered to an abuser. Other reasons for not wanting support from others might be related to feelings of loyalty and obligations in caring for the person, but also because the family member might not feel safe with the formal support or that the person with dementia refuses support. This highlights the need for early, individualised, and accessible support including information and training for family caregivers [32]. Also, to encourage families to ask for and accept offered support, it is important to build trustful relationships between health/social care staff and family members.

Some witnessed acts indicated that the person with dementia was exposed to multiple forms of elder abuse, that is, a form of polyvictimisation. Polyvictimisation is highly negatively associated with physical and psychosocial health [7], making it important that staff have knowledge and are aware of the signs of different types of abuse. When working with victims of elder abuse and not least when polyvictimisation occurs, a socio-ecological model can be a supportive framework, in particular because it highlights the complex interplay between risk factors at different social levels. Also, the model could explain why several interventions and comprehensive care might be needed to support an abused person [33].

### Limitations

The CSQ instrument has not been validated; however, the developers argue that assessment of performance using vignettes has demonstrated acceptable validity in comparison with performance in real consultations [22], and that content validity has been demonstrated through professional consensus [27]. One limitation in using group interviews is that some participants might find it difficult to voice their opinion freely if the topic is sensitive. Also, two of the interviews were performed digitally which risks affecting the interaction negatively. This was a convenience choice made as all participants in this group were familiar with video calls and lacked time to meet in-person. Using (digital) individual interviews could have been an alternative to make the participants feel more safe. However, the advantage of group members interacting with each other would be missed out if (digital) individual interviews had been used. Using a vignette helped participants to recall their own examples of difficult situations that can arise and lead to abuse. The Swedish version of the CSQ was translated by the researchers, and a limitation is that no back translation was made. The staff reviewing the translations received both versions A and B of the CSQ. Fewer comments were received regarding the language and meaning of the strategies in version B. Therefore, the authors decided to use that version even though it has been used less often in other studies. Furthermore, the sample size was small and there were missing data in some of the strategies. Consequently, no general conclusions should be drawn from the CSQ.

Performing a deductive analysis means that there will be content in the interviews that does not fit into the chosen categories. Also, there is no standard formula or ‘right’ way of doing the analysis [28] or interpreting each type of abuse. For the analysis, a chart describing the most common types of elder abuse [6] was used. However, it was sometimes difficult to determine where to put different witnessed acts as they can be related to several different types of abuse. For the validity of the study, ambiguities were discussed in the research group. Participants described situations in which the person with dementia was the abuser rather than the abused person, as well as situations in which a professional was the abuser. However, in the present study only witnessed abusive acts against the person with dementia were included because the instrument (CSQ) focused on abuse of the person with dementia [22, 27].

## Conclusions

Participants described several situations and different types of abuse they have encountered when working with persons with dementia, indicating that health and social care has the ability to detect abuse perpetrated by family members. However, inconsistencies in defining abusive behaviour management strategies demonstrate the uncertainty in identifying abuse. This may lead to abuse not being identified, but it also creates feelings of inadequacy among staff.

There is large variation among health and social care staff training regarding elder abuse and educational interventions, and reflections might be useful to increase knowledge and awareness. However, further research is needed, especially due to the difficulties in defining abuse among older persons with dementia.
